# Knowledge and Awareness of Pre‐Exposure Prophylaxis Among Men in Sub‐Saharan Africa: A Scoping Review Protocol

**DOI:** 10.1002/hsr2.70377

**Published:** 2025-01-27

**Authors:** Oluwaseun Badru, Mbuzeleni Hlongwa, Oluwafemi Atanda Adeagbo

**Affiliations:** ^1^ Department of Community and Behavioral Health University of Iowa Iowa City Iowa USA; ^2^ College of Health Sciences School of Nursing and Public Health, University of KwaZulu‐Natal Durban South Africa; ^3^ Public Health, Societies and Belonging Human Sciences Research Council Pretoria South Africa; ^4^ Department of Sociology University of Johannesburg Johannesburg South Africa

**Keywords:** awareness, knowledge, men, PrEP, sub‐Saharan Africa

## Abstract

**Introduction:**

About 39.9 million people were living with HIV as of 2023, and HIV is more prevalent in sub‐Saharan Africa. Pre‐exposure prophylaxis (PrEP) is highly effective in HIV prevention. Despite the efficacy of PrEP, many persons, including men, do not have adequate knowledge and awareness of PrEP, and reviews on knowledge and awareness among men are scarce. This review aims to assess and synthesize the knowledge and awareness of PrEP among persons assigned as males at birth in sub‐Saharan Africa.

**Methods and Analysis:**

The proposed scoping review will be conducted in accordance with the PRISMA Extension for Scoping Reviews (PRISMA‐ScR): Checklist and Explanation. The following information sources will be searched to retrieve relevant studies for this review: CINAHL, MEDLINE (Ovid), PubMed, SCOPUS, and Web of Science. Google Scholar, The Union Catalogue of Theses and Dissertations (UCTD) and SA ePublications via SABINET Online, WorldCat Dissertations and Theses via OCLC, ResearchGate, and American Doctoral Dissertations via EBSCOhost. All study designs, except existing reviews, will be included. All screenings (abstract screening and full‐text screening) and data extraction will be conducted independently by two reviewers. Quantitative findings will be presented with frequency and percentages, while qualitative thematic analysis will be used to analyze qualitative findings.

**Conclusion:**

This study will map out studies on knowledge and awareness of PrEP among men in sub‐Saharan Africa. The results of this review will give insights into what men in sub‐Saharan Africa know about PrEP, which can inform future interventions.

## 1 Introduction

About 39.9 million people were living with HIV as of 2023, which is more prevalent in sub‐Saharan Africa [1]. Abstinence, appropriate condom use, and biomedical prevention such as post‐exposure prophylaxis (PEP) and pre‐exposure prophylaxis (PrEP) are strategies to prevent HIV [2]. In 2016, the World Health Organization (WHO) recommended PrEP to priority groups, such as transgender people, sex workers (SWs), and men who have sex with other men (MSM) [3].

PrEP is a biomedical HIV prevention in the form of daily oral, on‐demand, or injectable medication to prevent HIV [4, 5]. PrEP has provided the opportunity to limit HIV transmission and, in turn, reduce incidence among priority groups and the general population [6, 7]. Oral PrEP is highly effective in HIV prevention [8, 9, 10]. PrEP, when taken at least 4 out of 7 days, reduces HIV risk by 75% to 99% [5, 11, 12]. Similarly, empirical studies have also shown that on‐demand PrEP (i.e., PrEP use before and after intercourse) [13, 14, 15] and injectable PrEP [16, 17] are effective in HIV prevention. However, the low perceived risk of HIV and poor awareness and knowledge of PrEP limits the use of PrEP, especially in HIV hyperendemic settings [12, 18].

Despite the efficacy of PrEP, many persons, including men (defined as those assigned male at birth: cisgender men and MSM), at risk of HIV do not have adequate knowledge or are not aware of PrEP. For example, only 51.5% of MSM surveyed in Kenya in 2020 were aware of PrEP [19]. Similarly, in 2022, only 45.8% of MSM and transgender women were aware of PrEP in Zimbabwe [11], and less than half (47.7%) of MSM in Rwanda were aware of PrEP as of 2021 [20].

Several individual and interpersonal factors are associated with awareness and knowledge of PrEP among men; individual factors such as self‐efficacy, having health insurance, and residing in an urban area determine awareness and knowledge of PrEP [21, 22]. Interpersonal factors such as having social support, being a member of a lesbian, gay, bisexual, and transgender organization, and having a partner who is living with HIV are associated with PrEP awareness [21, 22, 23].

Generally, men are less likely to know their HIV status and are more likely to be unengaged in HIV care than women [24]. For instance, 90% of women in South Africa are aware of their HIV status compared to 82% of men, and more women are on antiretroviral therapy than men (65% vs. 54%) as of the end of 2016 [24, 25]. This suggests that men are behind in seeking care HIV care and prevention due to multiple interpersonal and structural factors, including long waiting hours at the clinics, stigma, financial constraints due to traveling costs, discrimination, privacy concerns, poor knowledge of PrEP, and where to assess it [26, 27].

Earlier reviews on PrEP have focused on willingness, uptake, adherence, barriers, and facilitators to PrEP among MSM [28, 29, 30, 31, 32]. The reason for the focus on MSM is understandable, as MSM are disproportionately more likely at risk of HIV, and the initial focus of PrEP was on priority groups, including transgender persons and MSM [3]. Kamitani et al. [29], in their scoping review, found that most studies on PrEP focused on MSM; the recommendation of PrEP for MSM by the WHO may have contributed to the focus on MSM [33]. However, we found no prior reviews specifically focused on men's knowledge and awareness of PrEP, irrespective of sexual orientation. To bridge this gap, this review aims to map evidence on knowledge and awareness of PrEP among men in sub‐Saharan Africa.

## 2 Methods and Analysis

The proposed scoping review will be conducted following the Arksey and O'Malley framework [34], and reported with the PRISMA Extension for Scoping Reviews (PRISMA‐ScR) [35].

1. Identifying the research questions,
2. Identifying relevant studies,
3. Study selection,
4. Charting the data,
5. Collating, summarizing, and reporting results.

### 2.1 Research Questions

- Are men in sub‐Saharan Africa knowledgeable and aware of PrEP?
- What are the associated factors with knowledge and awareness of PrEP among men in sub‐Saharan Africa?

### 2.2 Identifying Relevant Studies

The search strategy aims to find both published and unpublished studies. We will conduct a preliminary search on PubMed, after which we will analyze text words and index terms used to describe the articles. This will assist in the creation of a search strategy that is specific to each information source. The following information sources will be searched to retrieve relevant studies for this review: CINAHL, MEDLINE (Ovid), PubMed, SCOPUS, and Web of Science. Google Scholar, The Union Catalogue of Theses and Dissertations (UCTD) and SA ePublications via SABINET Online, WorldCat Dissertations and Theses via OCLC, ResearchGate, and American Doctoral Dissertations via EBSCOhost will all be searched for unpublished academic research (theses and/or dissertations). All studies eligible for inclusion will have their reference lists checked for potential additional articles. Our search technique will include Boolean terms (AND, OR) and Medical Subject Headings (MeSH) phrases. The following key search words will be used: knowledge, awareness, PrEP (and specific medication such as Truvada and Descovy), men, and sub‐Saharan Africa. Some relevant MeSH terms include “Health Knowledge, Attitudes, Practice”[Mesh], “Awareness”[Mesh], “Pre‐Exposure Prophylaxis”[Mesh], “Men”[Mesh], and “Africa South of the Sahara”[Mesh]. Each keyword will be combined with relevant MeSH and synonymous terms with the “OR” Boolean operator to form a block, while each block will be combined with the “AND” Boolean operator (see Supporting Information S1: Table). We will also employ African country names and abbreviated terms like “East Africa/West Africa” to ensure that articles indexed using African nation‐specific names or regional terms are retrieved.

### 2.3 Eligibility Criteria

#### 2.3.1 Types of Participants

Only studies focused on adults (18 years and above) assigned male at birth (heterosexual men and MSM) will be included.

#### 2.3.2 Type of Outcome

Studies that assessed knowledge or awareness of PrEP (daily oral, on‐demand, and injectable) among men in sub‐Saharan Africa will be considered.

#### 2.3.3 Type of Studies

This scoping review will include all study designs except for existing reviews (rapid review, scoping review, systematic review with or without meta‐analysis, among others). However, only the baseline findings of cohort studies and trials (randomized or non‐randomized trials) will be reported. Studies with disaggregated data for men will also be included. Furthermore, conference proceedings and relevant dissertations will be considered in this review.

#### 2.3.4 Location

Only studies published in sub‐Saharan Africa will be considered in this review.

#### 2.3.5 Other Criteria

Studies published earlier than 2012 will be excluded since the first PrEP drug was approved in the year 2012 [36]. There will be no restriction on language.

### 2.4 Study Procedure and Selection of the Studies

All citations identified by the search will be downloaded and imported into the Rayyan systematic review manager for deduplication and screening of articles [37]. Two authors (O.A.B. and M.H.) will screen the title and abstract of all articles independently to determine their eligibility. Articles that do not meet the study's eligibility criteria will be excluded. The full text of articles that appear to have met the eligibility criteria will be obtained and screened independently by two authors (O.A.A. and O.A.B.). Discussion or collaboration with a third reviewer will be used to settle any disputes between the two independent reviewers, if any.

### 2.5 Charting the Data

The Joanna Briggs Institute's template for data extraction will be adapted for data extraction. The following data will be extracted from each study that meets the eligibility criteria: last name of the first author, year of publication, study objective(s), study setting, participant's characteristics, sample size, the country where the study was conducted, sampling method, data collection method, data analysis, study limitations and implications, knowledge and/or awareness of PrEP (in the form of prevalence or responses to individual questions), factors associated with knowledge and awareness of PrEP (bivariate and multivariate analysis), and conclusions from authors. Two reviewers will also independently extract data from the articles (O.A.B. and M.H.). Discussion with a third reviewer will settle any disputes.

### 2.6 Collating, Summarizing, and Reporting the Results

Quantitative findings will be synthesized and summarized with a narrative approach. The prevalence of knowledge and awareness will be descriptively summarized. Furthermore, descriptive statistics will be used to represent the region based on the WHO regional classification, sampling method (probability or non‐probability), characteristics of the participants, and factors associated with knowledge and awareness of PrEP. Qualitative thematic analysis will be used to analyze qualitative studies. Depending on the findings, the quantitative and qualitative findings will be triangulated.

### 2.7 Patient and Public Involvement

Patients or the public will not be directly involved in designing or disseminating this review's findings.

### 2.8 Dissemination

The findings of this review will be published in an open‐access journal for adequate dissemination and publicity. They will also be shared with relevant researchers, policymakers, and nongovernmental organizations interested in HIV prevention.

### 2.9 Ethical Consideration

Ethical clearance is not warranted as already published studies will be reviewed and synthesized.

## 3 Discussion

This scoping review will assess the knowledge and awareness of PrEP and associated factors among men in sub‐Saharan Africa. This review will be the first to chart PrEP knowledge of men in sub‐Saharan Africa. This review will help understand the level of PrEP knowledge among men in sub‐Saharan Africa and the factors that predict PrEP knowledge. The identified predictors or determinants can serve as targets for future interventions to improve knowledge and awareness of PrEP among men. Furthermore, the findings of this review may lead to policy formation tailored toward men's knowledge in several sub‐Saharan African countries. The findings of this review will be disseminated in the form of conference presentations and peer‐review publications. We anticipate some limitations. Some studies may have poor method quality since non‐peer‐reviewed publications will be considered, impacting the findings. Moreover, the findings may not be generalizable because the included studies will be largely observational, and causal inference cannot be established.

## Author Contributions

**Oluwaseun Badru:** conceptualization, methodology, writing–original draft, writing–review and editing, formal analysis, data curation. **Mbuzeleni Hlongwa:** writing–original draft, conceptualization, methodology. **Oluwafemi Atanda Adeagbo:** writing–original draft, conceptualization, methodology, supervision.

## Conflicts of Interest

The authors declare no conflicts of interest.

## Transparency Statement

The lead author, Oluwafemi Atanda Adeagbo, affirms that this manuscript is an honest, accurate, and transparent account of the study being reported, that no important aspects of the study have been omitted and that any discrepancies from the study as planned (and, if relevant, registered) have been explained.

## Supporting information

Supporting information.

## Data Availability

The authors have nothing to report.
