# Supplementary material for: Knowledge and Awareness of Pre‐Exposure Prophylaxis Among Men in Sub‐Saharan Africa: A Scoping Review Protocol
Source: Health Sci Rep. 2025 Jan 27;8(1):e70377. doi: 10.1002/hsr2.70377 (PMC11772214; doi:10.1002/hsr2.70377)
Supplement: Supplementary file 1 — Supporting information. [file HSR2-8-e70377-s001.docx]

**Table S1: Search strategy on PubMed**

| **SEARCH** | **KEY WORD AND MeSH terms** | **HIT** |
| --- | --- | --- |
| #1 | "Health Knowledge, Attitudes, Practice"[Mesh] OR "Awareness"[Mesh] OR knowledge OR awareness | [1,231,1](https://pubmed.ncbi.nlm.nih.gov/?term=%22Health+Knowledge%2C+Attitudes%2C+Practice%22%5BMesh%5D+OR+%22Awareness%22%5BMesh%5D+OR+knowledge+OR+awareness&size=200&sort=relevance)45 |
| #2 | "Pre-Exposure Prophylaxis"[Mesh] OR “pre-exposure prophylaxis" OR PrEP OR Truvada OR Descovy | [15,151](https://pubmed.ncbi.nlm.nih.gov/?term=%22Pre-Exposure+Prophylaxis%22%5BMesh%5D+OR+%E2%80%9Cpre-exposure+prophylaxis%22+OR+PrEP&size=200&ac=no&sort=relevance) |
| #3 | "Men"[Mesh] OR men OR “men who have sex with men” OR MSM OR Gay | [642,862](https://pubmed.ncbi.nlm.nih.gov/?term=%22Men%22%5BMesh%5D+OR+men+OR+%E2%80%9Ctransgender+wom%2A%E2%80%9D+OR+%E2%80%9Ctrans+wom%2A%E2%80%9D+OR+%E2%80%9Cmen+who+have+sex+with+men%E2%80%9D+OR+MSM+OR+Gay&size=200&ac=no&sort=relevance) |
| #4 | "Africa South of the Sahara"[Mesh] OR “sub-Saharan Africa” OR Angola OR Benin OR Botswana OR “Burkina Faso” OR Burundi OR Cameroon OR “Cape Verde” OR “Central African republic” OR Chad OR Comoros OR Congo OR “Cote d’Ivoire” OR Djibouti OR Eritrea OR Gabon OR Gambia OR Ghana OR Guinea OR Kenya OR Lesotho OR Liberia OR Madagascar OR Malawi OR Mali OR Mauritania OR Mauritius OR Mozambique OR Namibia OR Niger OR Nigeria OR Rwanda OR “Sao Tome” OR Principe OR Senegal OR Seychelles OR “Sierra Leone” OR Somalia OR “South Africa” OR Sudan OR Swaziland OR Tanzania OR Togo OR Uganda OR Zambia OR Zimbabwe OR “West Africa” OR “East Africa” OR “Southern Africa” OR “Central Africa” | [668,](https://pubmed.ncbi.nlm.nih.gov/?term=%22Africa+South+of+the+Sahara%22%5BMesh%5D+OR+%E2%80%9Csub-Saharan+Africa%E2%80%9D+OR+Angola+OR+Benin+OR+Botswana+OR+%E2%80%9CBurkina+Faso%E2%80%9D+OR+Burundi+OR+Cameroon+OR+%E2%80%9CCape+Verde%E2%80%9D+OR+%E2%80%9CCentral+African+republic%E2%80%9D+OR+Chad+OR+Comoros+OR+Congo+OR+%E2%80%9CCote+d%E2%80%99Ivoire%E2%80%9D+OR+Djibouti+OR+Eritrea+OR+Gabon+OR+Gambia+OR+Ghana+OR+Guinea+OR+Kenya+OR+Lesotho+OR+Liberia+OR+Madagascar+OR+Malawi+OR+Mali+OR+Mauritania+OR+Mauritius+OR+Mozambique+OR+Namibia+OR+Niger+OR+Nigeria+OR+Rwanda+OR+%E2%80%9CSao+Tome%E2%80%9D+OR+Principe+OR+Senegal+OR+Seychelles+OR+%E2%80%9CSierra+Leone%E2%80%9D+OR+Somalia+OR+%E2%80%9CSouth+Africa%E2%80%9D+OR+Sudan+OR+Swaziland+OR+Tanzania+OR+Togo+OR+Uganda+OR+Zambia+OR+Zimbabwe&size=200&ac=no&sort=relevance)151 |
| #5 | #1 AND #2 AND #3 AND #4 | 76 |
